# Advanced design and development of nanoparticle/microparticle dual-drug combination lactose carrier-free dry powder inhalation aerosols

**DOI:** 10.1039/d0ra07203f

**Published:** 2020-11-17

**Authors:** Priya Muralidharan, Evan K. Mallory, Monica Malapit, Hanna Phan, Julie G. Ledford, Don Hayes, Heidi M. Mansour

**Affiliations:** The University of Arizona, College of Pharmacy 1703 E. Mabel St Tucson AZ 85721-0207 USA mansour@pharmacy.arizona.edu +1-520-626-2768; The Asthma & Airway Disease Research Center Tucson AZ USA; The University of Arizona College of Medicine, Department of Cellular & Molecular Medicine Tucson AZ USA; The Departments of Pediatrics and Internal Medicine, Lung and Heart–Lung Transplant Programs, The Ohio State University College of Medicine Columbus OH USA; The University of Arizona College of Medicine, Department of Medicine, Division of Translational & Regenerative Medicine Tucson AZ USA; The University of Arizona, The BIO5 Research Institute Tucson AZ USA; The University of Arizona, Institute of the Environment Tucson AZ USA

## Abstract

*Rationale*: lactose is the only FDA-approved carrier for dry powder inhaler (DPI) formulations in the US. Lactose carrier-based DPI products are contraindicated in patients with a known lactose allergy. Hence, inhaler formulations without lactose will benefit lactose allergic asthmatics. *Objectives*: to rationally design and develop lactose carrier-free dry powder inhaler formulations of fluticasone propionate and salmeterol xinafoate that will benefit people with known lactose allergy. The study also aims at improving the aerosol deposition of the dry powder formulation through advanced particle engineering design technologies to create inhalable powders consisting of nanoparticles/microparticles. *Methods*: advanced DPI nanoparticle/microparticle formulations were designed, developed and optimized using organic solution advanced closed-mode spray drying. The co-spray dried (co-SD) powders were comprehensively characterized in solid-state and *in vitro* comparative analysis of the aerodynamic performance of these molecularly mixed formulations was conducted with the marketed formulation of Advair® Diskus® interactive physical mixture. *Measurements and main results*: comprehensive solid-state physicochemical characterization of the powders showed that the engineered co-SD particles were small and spherical within the size range of 450 nm to 7.25 μm. Improved fine particle fraction and lower mass median aerodynamic diameter were achieved by these DPI nanoparticles/microparticles. *Conclusions*: this study has successfully produced a lactose-free dry powder formulation containing fluticasone propionate and salmeterol xinafoate with mannitol as excipient engineered as inhalable DPI nanoparticles/microparticles by advanced spray drying. Further, co-spray drying with mannitol and using Handihaler® device can generate higher fine particle mass of fluticasone/salmeterol. Mannitol, a mucolytic agent and aerosol performance enhancer, is a suitable excipient that can enhance aerosol dispersion of DPIs.

## Abstract 2

Advanced co-spray drying of fluticasone propionate, salmeterol xinafoate, and d-mannitol leads to high-performing inhalable dry powders as molecular mixtures.

## Graphical abstract

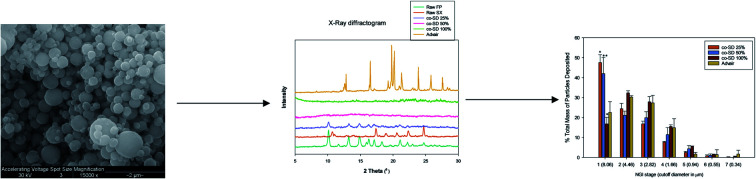

## Introduction

According to the World Health Organization (WHO), asthma and chronic obstructive pulmonary disease (COPD) affect 65 million and 325 million people worldwide, respectively.^1^ One of the most common of the respiratory diseases that afflicts both children and adults, asthma presents as a syndrome of non-specific airway hyperresponsiveness, inflammation and intermittent respiratory symptoms. While asthma onset can be triggered in some by infection, environmental allergens or other stimuli,^2,3^ asthma in others is associated more with genetic factors.^4^ With the advancement of our understanding of the newly described endotypes of asthma,^5^ asthma therapies are becoming more patient-targeted. Despite new endotype-targeted biologics, the mainstay of therapies spanning all endotypes remain the drugs that help to quickly expand the airway (bronchodilator) or control inflammation (ICS-LABA).^6,7^

Pulmonary drug delivery^8^ has become a sophisticated field presenting greater opportunities to achieve more efficient and targeted drug delivery.^9^ Since the introduction of the Montreal Protocol and the banishment of the use of chlorofluorocarbons (CFCs) as a propellant, dry powder inhalers (DPIs) have become an effective and the predominant inhalation class for many of the medications used to manage asthma and COPD.^10^ An advantage of DPIs include the lack of requiring patient coordination between device actuation and inhalation, allowing more effective delivery of the medication in the patient population who struggle with this aspect of pressurized metered dose (pMDI) inhaler use.^11,12^

Management of more persistent forms of asthma and later stages of COPD focus on the combined use of an inhaled corticosteroid (ICS) with an inhaled long-acting beta-agonist (LABA).^13,14^ Advair® Diskus® (GlaxoSmithKline), which is an example of this combined pharmacotherapy, was introduced to the United States drug market in the 2000s as the first dual-drug product for the maintenance treatment of asthma in patients of age 12 years and older. Since then, it has been approved for the management of asthmatics for ages four and older as well as, treatment of COPD patients. As a dual-drug combination marketed product containing both fluticasone propionate (FP) and salmeterol xinafoate (SX), Advair® Diskus® was the first dual-drug DPI product to simultaneously treat both inflammation and bronchoconstriction in asthma and COPD patients. This inhaler was shown to improve patients' management of their disease state and achieved synergism which was not seen in using two separate inhalers.^15^ The Diskus® inhaler device has been and continues to be well-received among patients for its ease of use and reliable performance.^16^

**Fig. 1:**
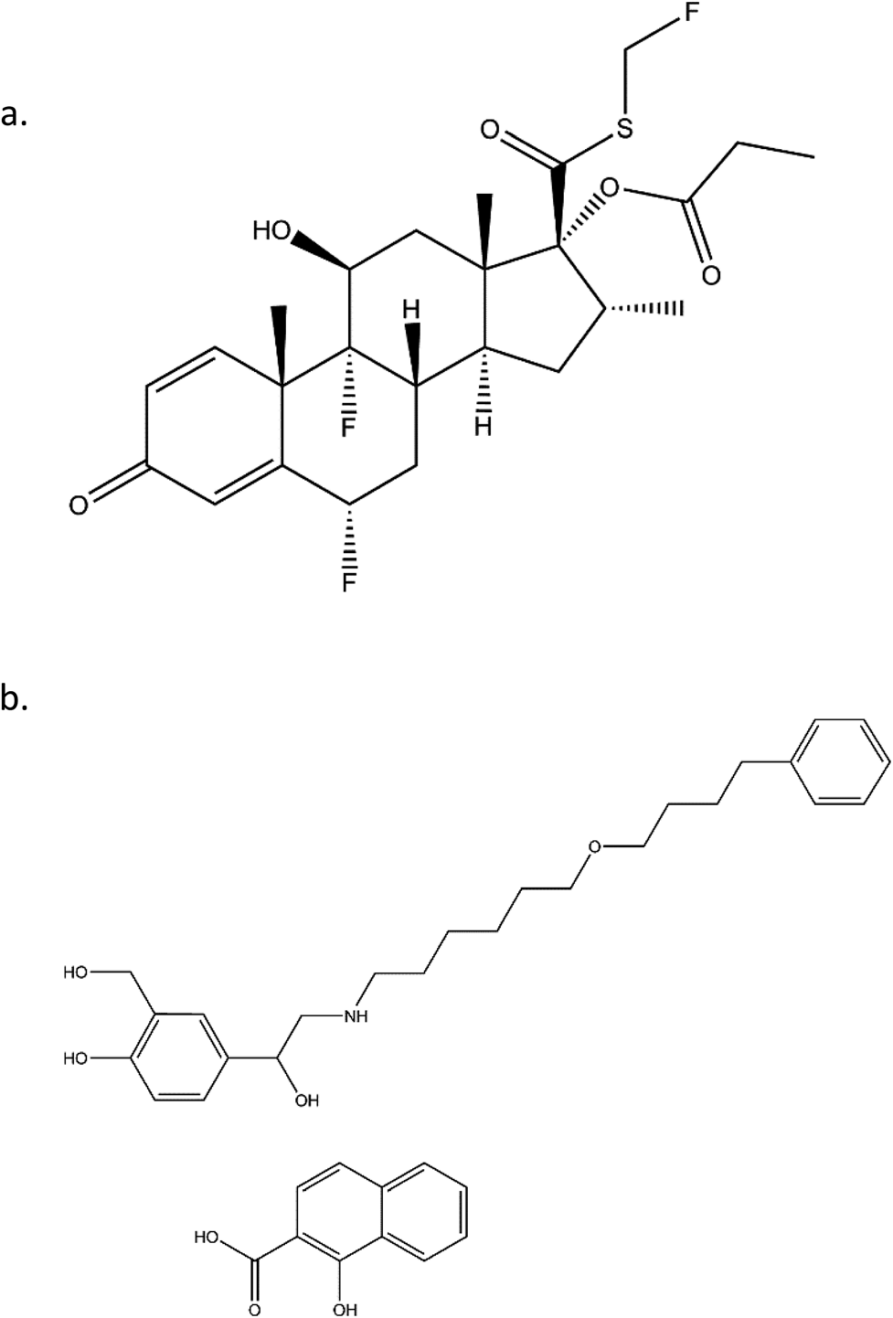
Chemical structures of: (a) Fluticasone Propionate (FP); and (b) salmeterol (SX).

FP, a corticosteroid derivative, is tri-fluorinated drug and is conjugated to propionic fatty acid, as shown in Fig. 1. Both of these properties contribute to its high hydrophobicity and enhance its ability to insert into membranes of the lung tissue and thereby allowing for long residence times. Similarly, SX has a long, hydrophobic tail that allows for long residence times. Beyond the structural characteristics that contribute to their long-acting pharmacokinetics, SX and FP are from distinct therapeutic drug classes, being LABA and ICS, respectively. It has been previously demonstrated that the delivery of these two agents from the same inhaler device has synergistic effects that are not apparent when delivered from separate devices.^15^

Compared to micronization techniques that are currently used to prepare the powder formulation in the dry powder inhaler products, spray drying offers several advantages because it allows for precisely engineered formulation of drug products that meet the desired particle characteristics through the adjustment of numerous process parameters (*i.e.* flow rate, temperature, solution concentration *etc.*). Spray dried formulations have been shown to achieve improved dispersibility, incorporate nanomedicine,^17–19^ and deep lung deposition than other formulation techniques.^20^ In recent years, DPI technologies have successfully adapted spray drying process to the advanced design and manufacture dry powder formulations with desired tailored solid-state physical chemical properties^21^ for respiratory drug delivery such as PulmoSol™, PulmoSpheres® technology by Nektar Therapeutics and Technosphere®. In general, particles with a smaller aerodynamic diameter have a greater ability to efficiently reach deeper within the smaller airways in the lung.^22,23^ Particles with an aerodynamic size in the range of 5–10 microns can enter the lungs and deposit in the larger airways (up to the first several divisions of the bronchi). Particles having less than 5 microns aerodynamic size can effectively reach the respiratory bronchioles of the mid-lung region and particles less than 2 microns aerodynamic size can effectively target the alveoli. By manipulating particle size, different areas of the lung can be targeted through tailoring to achieve optimal drug delivery for specific respiratory disease states.^24^

The currently approved drug formulation within the Advair® Diskus® inhaler is a micronized drug particles of FP and SX physically blended large lactose monohydrate carrier particles as an excipient to create an interactive physical mixture (*i.e.* a blend).^25–27^ The micronized drug particles are created by air jet-milling to render them into the respirable size range necessary for lung deposition following inhalation. The large lactose monohydrate carrier particles are typically in the size range of 75–120 microns and reach the gastrointestinal (GI) tract by swallowing following inertial impaction^23,28^ in the oropharyngeal region. Because Advair® is a lactose-containing product, the use of it and other DPIs containing lactose are contraindicated in patients with a known lactose allergy.^29^ Furthermore, the population rates of milk protein (*e.g.* casein, albumin, and whey) allergy are estimated to affect 1.2% to as much as 17% of people of all ages, thus limiting the utility of this product and other lactose-containing DPIs in this population.^30^

The purpose of this systematic and comprehensive study is to rationally design and develop a lactose carrier-free formulations of FP/SX that will have no immunogenic response and to create dual-drug DPIs as inhalable nanoparticles and microparticles. Several other approaches to lactose carrier-free dry powder inhaler formulations have been reported.^31^ However, this study reports for the first time on advanced co-spray dried (co-SD) FP/SX with mannitol (Man) excipient as molecular mixtures under these advanced organic solution spray drying conditions using advanced particle engineering design technologies. These are designed as inhalable powders comprised of nanoparticles/microparticles. Mannitol, a pharmaceutical excipient, has been previously shown by us to have uniquely favorable properties to improve the aerosol properties of therapeutic dry powder inhalation aerosols.^32–34^ In addition, the co-SD particles were comprehensively characterized and *in vitro* comparative analysis of aerodynamic performance of these molecularly mixed formulations with the marketed formulation of Advair® Diskus® 250/50 (FP/SX) interactive physical mixture of jet-milled micronized respirable drugs blended with large non-respirable lactose monohydrate carrier particles was also examined. To the authors' knowledge, we are the first to report on these studies using these conditions.

## Materials and methods

### Materials

Fluticasone Propionate (FP) and Salmeterol Xinafoate (SX) were obtained from APAC pharmaceuticals LLC. d-Mannitol was obtained from Acros Organics. Methanol (HPLC grade, ACS-certified grade, purity 99.9%) was obtained from Fisher Scientific. Hydranal®-Coulomat AD was obtained from Sigma-Aldrich. The nitrogen gas used was ultra-high purity (UHP) nitrogen gas. All solvents used were ACS/HPLC grade. After spray drying, the powders were stored individually in sealed glass desiccators over Indicating Drierite™ desiccant at −20 °C. Chemical structures were drawn using ChemBioDraw® Ultra Version 14.0.0117 software.

### Methods

#### Preparation of co-spray dried (co-SD) particles by organic solution advanced spray drying in closed mode

Organic solution advanced spray drying was carried out similar to the method previously mentioned.^33^ The feed solution contained 3 : 1 API (5 : 1 FP : SX) : mannitol in molar ratio dissolved in methanol using ultrasonication. Table 1 lists the spray drying conditions of the powders. Three feed pump rates low, medium and high were used to produce the co-SD particles.

**Table 1:** Organic solution advanced spray drying conditions in closed-mode

Feed pump rate (PR)%	25% (7.5 mL min^−1^)	50% (15 mL min^−1^)	100% (30 mL min^−1^)
Inlet temperature (°C)	130	130–131	129–131
Outlet temperature (°C)	80–81	61–65	35–47
Aspirator rate (m^3^ h^−1^)	37.5	37.5	37.5
Atomization gas flow rate (L h^−1^)	601	601	601
Percent yield (%)	56.78	59.03	70.15

#### Scanning electron microscopy (SEM) and energy dispersive X-ray (EDX) spectroscopy

Using conditions similar to previously reported,^32,35–37^ all co-SD FP/SX/Man particles and the Advair® physical mixture were imaged and analyzed to determine particle size, morphology, and surface morphology using SEM. The particles were gold coated for 90 s to assist the image capture using an electron beam of 30 kV accelerating voltage. Elemental analysis of the powder samples was performed using EDX with the spot size adjusted to attain a dead time of 20–30.

#### Particle sizing and size distribution using SEM micrographs

The particle size distribution was determined from SEM micrographs at 10 000× using SigmaScan Pro 5.0.0, using similar conditions previously reported.^38^ Diameter of at least 100 particles per image per sample was measured.

#### X-ray powder diffraction (XRPD)

Using similar conditions as previously reported,^32,35–37^ X-ray diffraction patterns were collected at room temperature using Cu Kα radiation (45 kV, 40 mA, and *λ* = 1.5406 Å) with a scan rate of 2.00° per minute at ambient temperature.

#### Differential scanning calorimetry (DSC)

Thermal analysis and phase transition measurements were performed similar to the method previously reported.^32,35–37^ The samples were heated from 0.00 °C to 350.00 °C at a scanning rate of 5.00 °C min^−1^.

#### Karl Fisher titration (KFT)

Using conditions similar to previously reported,^32,35–37^ the residual water content was quantified by Karl Fisher titration (KFT) coulometrically using Hydranal® Coulomat AD reagent.

#### Confocal Raman microspectroscopy (CRM), chemical imaging, and mapping

Confocal Raman microspectroscopy (CRM) provides noninvasive and nondestructive microspectroscopic component analysis of DPI formulations. Using similar conditions as previously reported.^32,33,35^ Raman spectra was obtained at 514 nm laser excitation using a 20× objective. Raman mapping was performed, similar to a previous study, in the range of 1052–2081 cm^−1^ with the spectrum centered at 1600 cm^−1^ wavenumber.^39^

#### Attenuated total reflectance (ATR)-Fourier transform infrared (FTIR) spectroscopy

The ATR-FTIR spectrum was collected with the ATR accessory similar to our previous studies.^32,35–37^ Briefly the conditions were 32 scans at a spectral resolution of 8 cm^−1^ over the wavenumber range of 4000–400 cm^−1^.

#### Drug content analysis by high performance liquid chromatography (HPLC)

High-performance liquid chromatography (HPLC) was used to quantify the amount of FP and SX in the co-SD powders. This method was performed with similar conditions reported by previously.^40,41^ Shimadzu LC-2010AHT HPLC system, with autosampler fitted to a 20 μL sampling loop and UV-Vis detector and reverse phase C_18_ phenomenex column with 250 × 4.6 mm, 5 μm particle size was used. The mobile phase consisted of 75 : 25 v/v methanol-0.6% (w/v) aqueous ammonium acetate solution degassed and run at a flow rate of 1.0 mL min^−1^. The column was maintained at 40 °C with a run time of 10 min. The injection volume was 20 μL. The UV detector was set to 228 nm, since both FP and SX had good absorption at this wavelength. The retention time of salmeterol was 5.7 min and fluticasone was 8.4 minutes. The retention peaks of xinafoic acid was seen around 3.09 min, but it was not used for quantification of the compounds. Different concentrations between 0.005–0.5 mg mL^−1^ was used for calibration curves of FP and SX respectively. All co-SD powders were dissolved at a known concentration (0.5 mg mL^−1^) and analyzed similar to the drug standards.

#### *In vitro* aerosol dispersion performance

In accordance with USP Chapter &lang;601&rang; specifications^42^ for pharmaceutical aerosol testing, Next Generation Impactor™ (NGI™) was used for the aerosol dispersion performance of all co-SD particles as detailed in previous reports.^32,35–37^ 10 mg of test powder was filled in Quali-V HPMC size 3 inhalation grade capsules and actuated using HandiHaler® (human DPI device) at 60 L min^−1^ (adult airflow rate). Three capsules were used for each run. For Advair® Diskus® particles, the preseparator was filled with 15 mL of purified water.

The emitted dose (ED) is the fraction of amount of powder released from the capsule following actuation of the inhaler to the total dose (TD) loaded in the capsule. The fine particle fraction (FPF) is the dose deposited on NGI stages 2 to 7 fine particle dose (FPD) over emitted dose (ED). The respirable fraction (RF%) eqn (3) was used as the percentage of fine particle dose to total deposited dose (DD) on all impactor stages.1
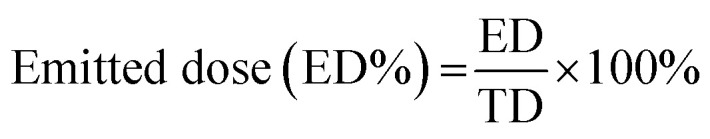
2
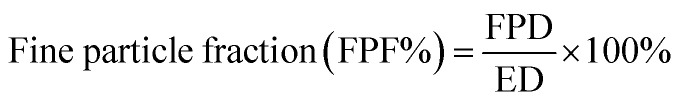
3
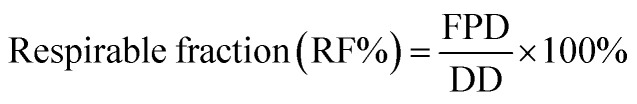


Wolfram Mathematica (Wolfram Research Inc., Champaign, Illinois) written by Dr Warren Finlay was used to calculate the mass mean aerodynamic diameter (MMAD) and the geometric standard deviation (GSD).

#### Statistical analysis

Unless otherwise mentioned, all analysis was performed in triplicate. Two-tailed Student *t*-test was used with a significance level (*p*-value) of ≤0.01 to determine statistical significance.

## Results

### SEM

**Fig. 2:**
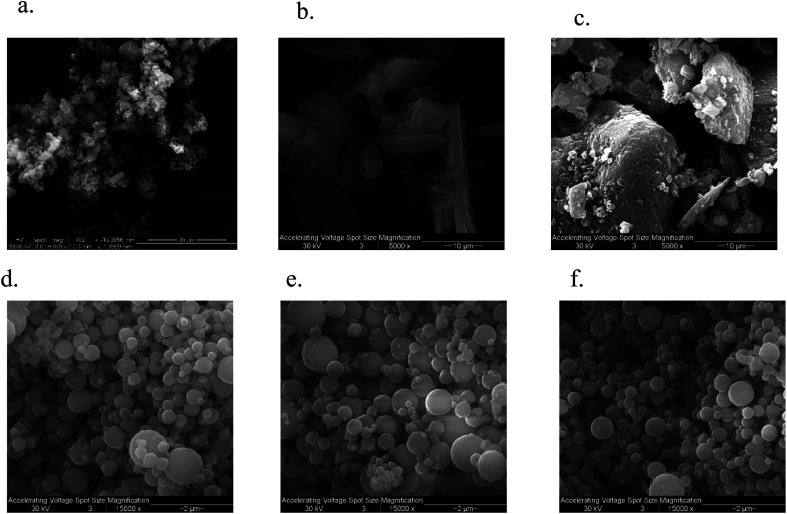
SEM micrographs of (a) raw fluticasone propionate (FP); (b) raw salmeterol xinafoate (SX); (c) Advair® Diskus® particles; (d) co-SD FP/SX/Man particles at 25% pump rate; (e) co-SD FP/SX/Man particles at 50% pump rate; and (f) co-SD FP/SX/Man particles at 100% pump rate.

The SEM micrograph of FP and SX before spray drying, Advair particles, and co-SD FP/SX/Man particles can be seen in Fig. 2. All co-SD particles were small, spherical with a smooth surface. The morphology of the co-SD particles was similar to each other while being different from that of the particles from Advair® Diskus®. This is due to spray drying which is well known to produce small spherical particles under the appropriate spray drying conditions.

### EDX spectrometry

**Fig. 3:**
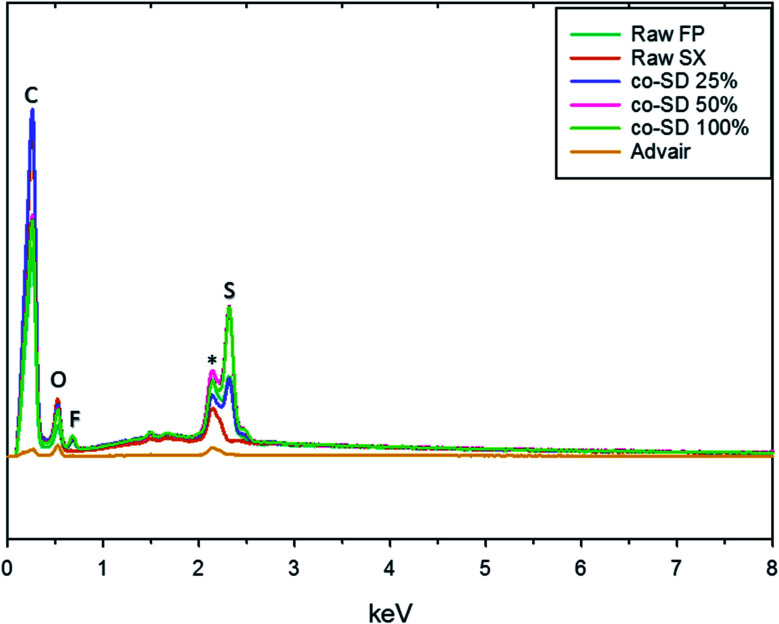
Energy Dispersive X-ray (EDX) of raw fluticasone propionate, raw salmeterol xinafoate, co-SD FP/SX/Man particles at 25% pump rate, co-SD FP/SX/Man particles at 50% pump rate, co-SD FP/SX/Man particles at 100% pump rate and Advair® Diskus® particles.

The EDX spectra seen in Fig. 3 shows the energy dispersion of unprocessed (raw) drugs, co-SD particles, and Advair® particles. The prominent peaks seen on the spectra were identified to be carbon, oxygen, fluorine, gold, and sulfur. It was difficult to identify nitrogen peak due to the energy overlapping with carbon and oxygen.

### Particle sizing and size distribution using SEM micrographs

Table 2 lists the measured particle size distribution of the co-SD FP/SX/Man particles from the SEM micrographs. The measured geometric particle size ranges from few hundred nanometers to 7 microns. The mean particle size range of the powders from different pump rates are 1.35, 1.22 and 1.27 μm for 25%, 50% and 100% PR respectively.

**Table 2:** Physicochemical properties of co-SD FP/SX/Mannitol particles

Powder composition	Spray drying pump rate%	Particle size range (μm)	Mean particle size (μm)	Residual water content (% w/w)	Mass of SX (mg mg^−1^ of SD powder)	Mass of FP (mg mg^−1^ of SD powder)
Co-SD FP : SX : Man	25	0.45–3.16	1.35	0.338 ± 0.046	0.1755 ± 0.037	0.5146 ± 0.061
Co-SD FP : SX : Man	50	0.35–3.51	1.22	0.676 ± 0.105	0.1599 ± 0.018	0.4401 ± 0.046
Co-SD FP : SX : Man	100	0.45–7.26	1.27	0.707 ± 0.066	0.1956 ± 0.023	0.5323 ± 0.058

### XRPD

**Fig. 4:**
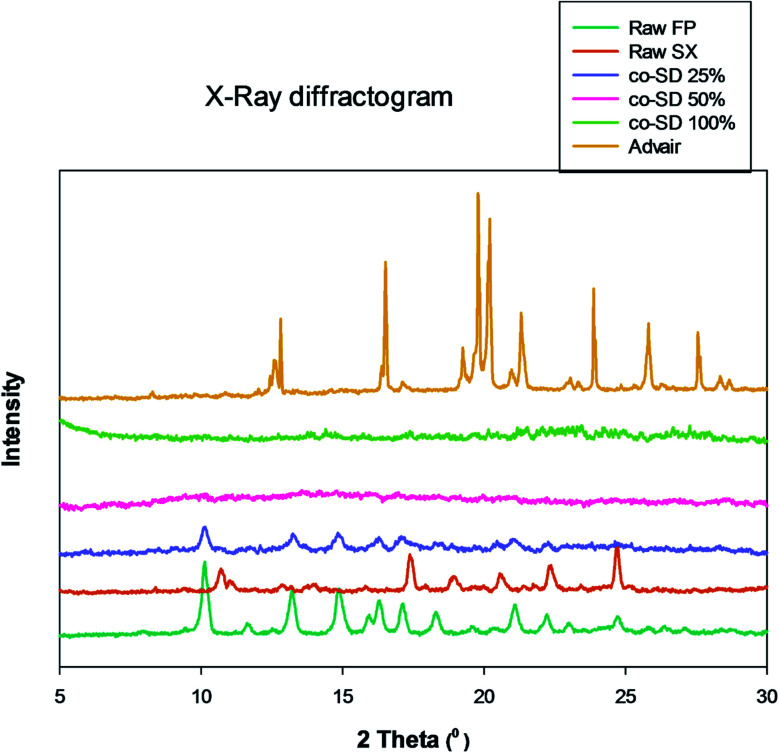
X-ray diffractogram of raw FP (before spray drying), raw SX (before spray drying), co-SD FP/SX/Man particles at 25% PR, co-SD FP/SX/Man particles at 50% PR, co-SD FP/SX/Man particles at 100% PR and Advair® particles.

Fig. 4 shows the X-ray diffractogram of FP, SX before spray drying, co-SD particles at different pump rates and Advair® particles. As can be seen in Fig. 4, the 2*θ* peaks were different in Advair® compared to the raw FP and SX. Co-spray drying of both drugs together with mannitol at 50% and 100% PR the particles were rendered non-crystalline as indicated by the absence of sharp diffraction peaks. On the other hand, the 25% PR exhibited slight crystallinity, which is evident from the diffraction peaks. Advair® particles exhibited sharp peaks characteristic of the crystalline state. Lactose XPRD diffractograms^43^ have been reported and may dominate in the Advair physical mixture, as it is present in a higher concentration than the two drugs.

### DSC

**Fig. 5:**
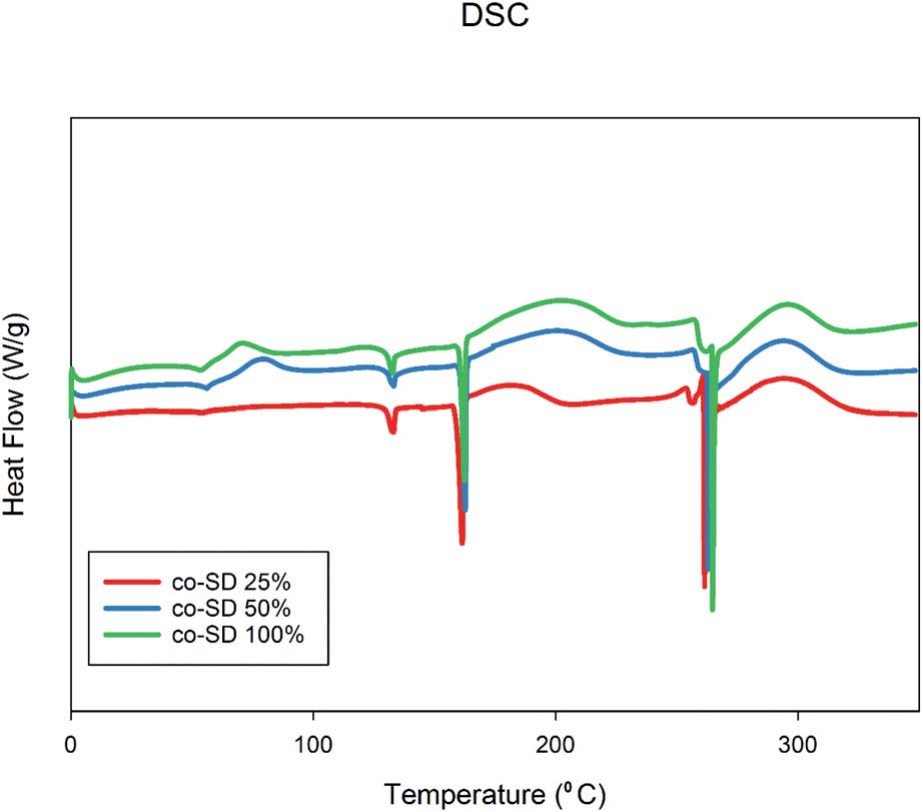
Differential Scanning Calorimetry thermographs of co-SD FP/SX/Man particles at 25% PR, co-SD FP/SX/Man particles at 50% PR, and co-SD FP/SX/Man particles at 100% PR.

Fig. 5 shows the thermograms of co-SD FP/SX/Man particles scanned over the temperature range of 0.00 °C to 350.00 °C. There were three endotherms observed in all powders which were consistent with the melting point of the three components of SX, Man and FP respectively. The transitions are consistent with the previously reports on SX,^27,44^ Man,^35^ and FP.^27,44^

### KFT

The residual water content measured in the co-SD FP/SX/Man particles at pump rate 25%, 50% and 100% were 0.34%, 0.68%, and 0.71% (w/w) respectively. All the powders had residual water content less than 1% (w/w).

### CRM, chemical imaging, and mapping

**Fig. 6:**
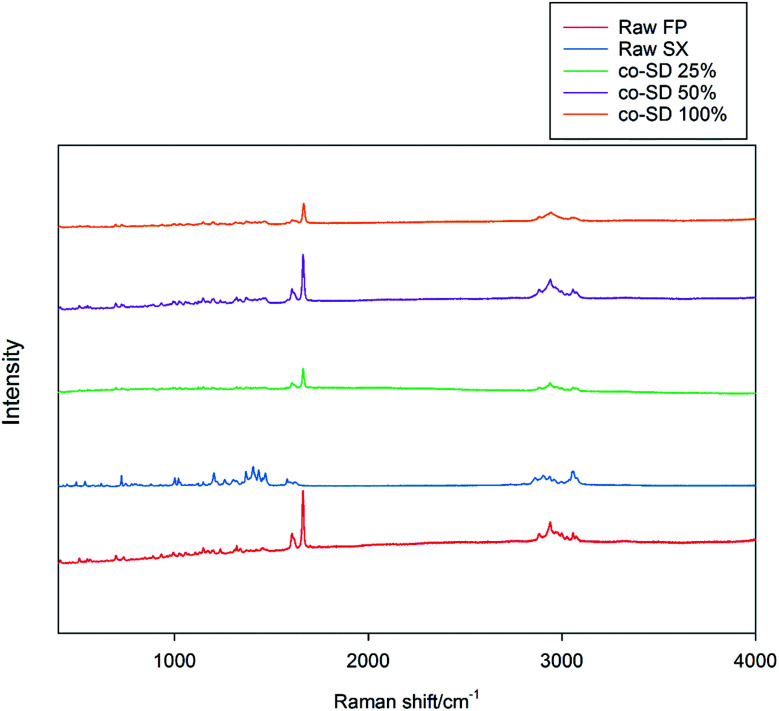
Raman Spectra of raw FP (before spray drying), raw SX (before spray drying), co-SD FP/SX/Man particles at 25% PR, co-SD FP/SX/Man particles at 50% PR, and co-SD FP/SX/Man particles at 100% PR.

**Fig. 7:**
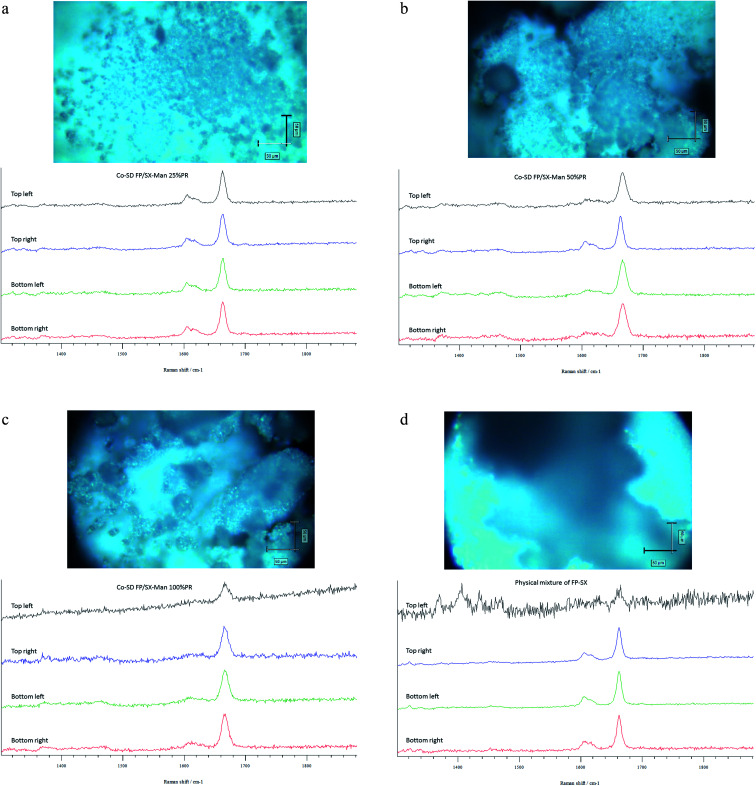
(a) Raman mapping of co-SD FP/SX/Man particles at 25% PR. (b) Raman mapping of co-SD FP/SX/Man particles at 50% PR. (c) Raman mapping of co-SD FP/SX/Man particles at 100% PR. (d) Raman mapping of blended physical mixtures of FP/SX jet-milled particles blended with large lactose monohydrate carrier particles.

The Raman spectra of all co-SD FP/SX/Man particles showed absorption peaks at 1607, 1664, 2882, 2940 and 3058 wavenumbers as seen in Fig. 6. The unprocessed (raw) FP had peaks at wavenumbers 1607, 1663, 2882, 2939 and 3057 in good agreement with previously published findings.^45^ Similarly, the major peaks of SX were found at 726,1001, 1204, 1369, 1469, 1581, 2905 and 3057 wavenumbers. The prominent peak of FP was seen at 1664 cm^−1^ for all co-SD FP/SX/Man particles. However, the peaks of SX were not distinctly seen in the co-SD systems. The Raman mapping images of the powders are seen in Fig. 7. A physical mixture of FP/SX was mapped using the same conditions. As it can be seen from Fig. 7d, the SX peak around 1400 wavenumbers were very faintly visible in the top left corner of the imaged powder sample.

### ATR-FTIR spectroscopy

**Fig. 8:**
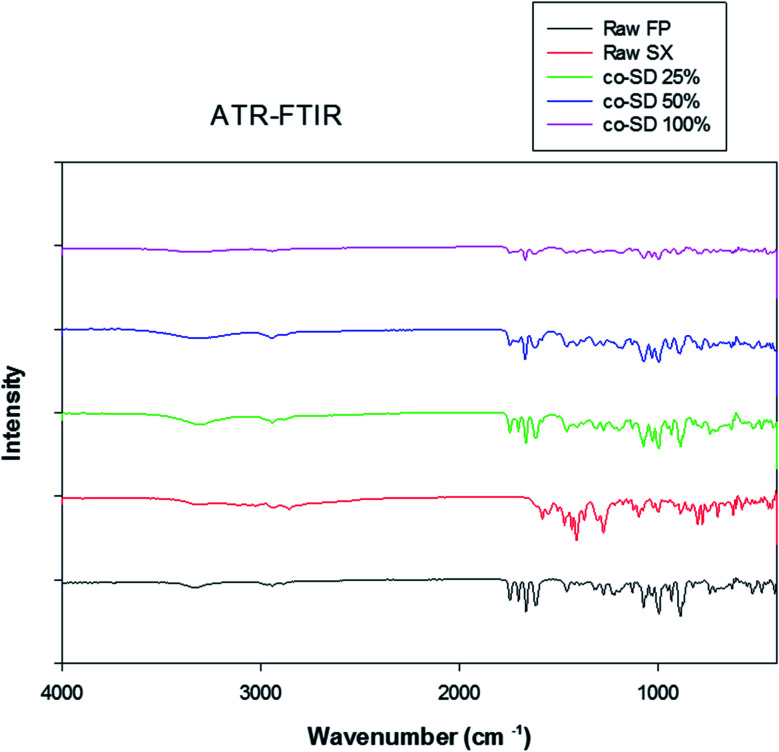
ATR-FTIR spectra of raw FP (before spray drying), raw SX (before spray drying), co-SD FP/SX/Man particles at 25% PR, co-SD FP/SX/Man particles at 50% PR, co-SD FP/SX/Man particles at 100% PR and Advair® particles.

The ATR-FTIR spectra of the unprocessed (raw) and co-SD drugs can be seen in Fig. 8. The FTIR spectra of FP shows OH stretching mode near 3330 cm^−1^ suggestive of external hydrogen bonding. The absorption bands observed in the fingerprint region of FP and SX were similar to previous reports.^45,46^ However, the absorption pattern of co-SD particles at all three pump rates resembles closely to FP than SX. This is most likely due to the increased ratio of FP to SX in the molecular mixed formulation.

### Drug content analysis by HPLC

**Fig. 9:**
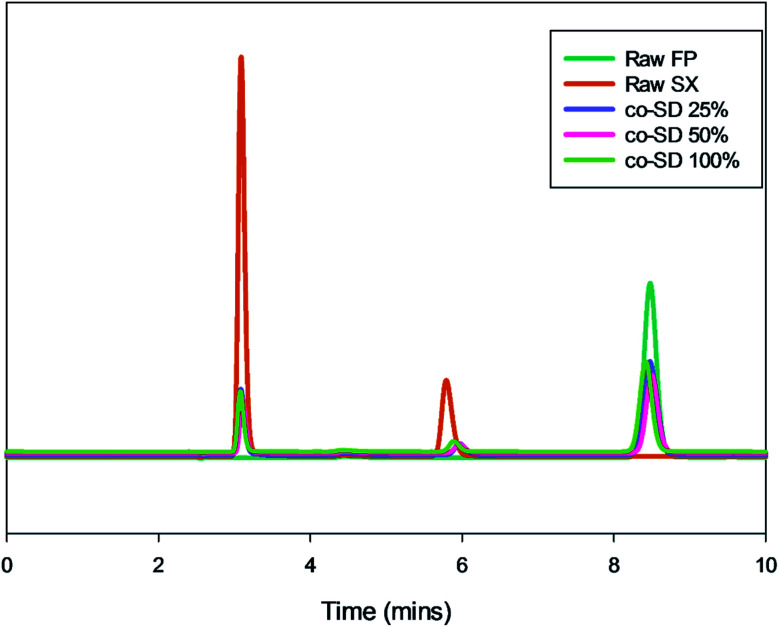
Representative HPLC chromatogram showing xinafoic acid (*t*_R_ = 3.09 min), salmeterol (*t*_R_ = 5.7 min), and fluticasone (*t*_R_ = 8.4 min).

The chromatographic method provided a good resolution of xinafoic acid (XA), salmeterol base, and Fluticasone Propionate as seen from Fig. 9. The calibration curves of FP and SX showed linearity (*r*^2^ ≥ 0.9979). The drug quantification results are listed in Table 2. Based on the calibration curve of the drug standards, the concentration of the drugs (Table 2) were SX 0.1755, 0.1599, and 0.1956 mg mg^−1^ at 25%, 50% and 100% PR respectively. Similarly, FP was quantified to be 0.5146, 0.4401, and 0.5323 mg mg^−1^ at 25%, 50% and 100% PR respectively.

### *In vitro* aerosol dispersion performance

**Fig. 10:**
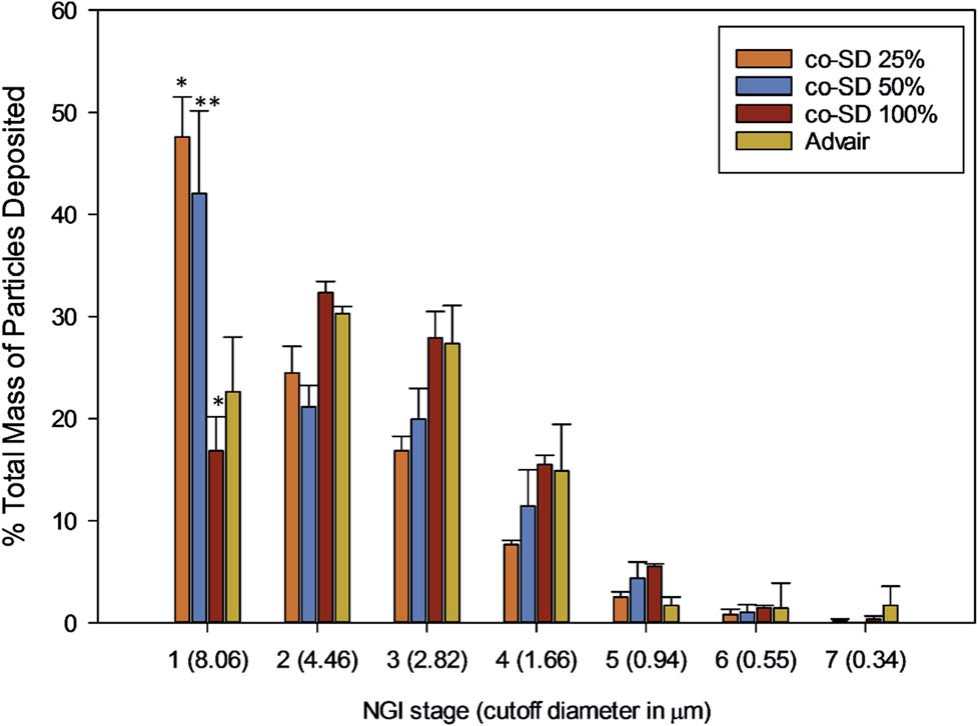
NGI stage deposition of co-SD FP/SX/Man particles at 25% pump rate, co-SD FP/SX/Man particles at 50% pump rate, co-SD FP/SX/Man particles at 100% pump rate and Advair® Diskus® particles.

The NGI stage deposition profile of Advair® and co-SD particles is presented in Fig. 10. Co-SD FP/SX/Man particles were capable of reaching the NGI stages 5–6, while Advair® particles were found to reach stage 7. Table 3 lists the *in vitro* aerosol dispersion performance of the co-SD FP/SX/Man particles. The FPF of the co-SD powders were 24%, 26% and 38% for 25% PR, 50% PR and 100% PR particles, respectively. The MMAD values of the powders were also found to be small in the range of 4.65 μm for high pump rate (100%) to 7.50 μm for low pump rate (25%). The fine particle mass of Advair® particles was calculated to be 3.22 mg, while that of the co-SD particles was in the range of 30–45 mg. All co-SD FP/SX/Man particles exhibited high ED values of ∼98% (w/w).

**Table 3:** *In vitro* aerosol dispersion performance with next generation impactor (NGI) using human DPI device Handihaler® for spray dried formulations compared to Advair® Diskus®^a^

Powder composition	Emitted dose (%)	Respirable fraction (%)	Fine particle dose (mg) fine particle fraction (%)	MMAD (μm)	GSD
SD FP/SX/Man 25%	97.18 ± 0.50	52.42 ± 22.87	10.13 ± 0.78	7.50 ± 3.26	2.27
			24.34 ± 0.23		
SD FP/SX/Man 50%	96.68 ± 0.69	57.95 ± 25.74	10.48 ± 0.11	6.14 ± 2.81	2.19
			26.11 ± 2.37		
SD FP/SX/Man 100%	97.53 ± 0.46	83.19 ± 36.10	15.13 ± 0.67	4.65 ± 2.02	1.96
			38.67 ± 1.39		
Advair® Diskus®	N/A	77.37 ± 33.72	10.00 ± 0.16	5.76 ± 2.83	2.50
			N/A		

a N/A: emitted dose and fine particle fraction were not calculated for Advair® Diskus® particles, since the total mass preloaded by the manufacturer for each actuation for this device was not known.

## Discussion

This study aims at developing a lactose carrier-free dry powder formulation of FP/SX that can benefit the patient population with known lactose allergy. In Advair® Diskus® formulation lactose serves two purposes, one is to bulk up the powder mass that must be inhaled by the patient and the other is to improve the aerosol dispersion by decreasing the high interparticulate interactions^47^ occurring between the small micronized drug particles. Previously, several techniques such as crystallization by sonication to make single inhalable crystal,^48^ co-precipitation,^44^ controlled crystallization^49^ of FP/SX followed by spray drying and blending with lactose were employed in development of the dual-drug DPI formulations of FP/SX. However, in the present study, FP/SX drug combinations were co-spray dried as molecular mixtures with mannitol. Chemically, mannitol is a non-reducing sugar with no known property to cause allergy, in contrast to lactose monohydrate. It can also stabilize the drug molecule in a solid-state matrix with a high transition temperature. Our lab has shown in the past that molecular mixtures with mannitol achieve superior aerosol performance,^32,33,35^ which is paramount for targeted delivery of therapeutics to the progressively converging airways. Additionally, mannitol is a mucolytic agent that can help in breaking the mucus blockage that is commonly seen in asthma and COPD patients. Hence, the use of mannitol in this formulation appears to be a more appropriate strategy for treating asthma.

Solid-state characterization of the co-SD formulations of FP/SX/Man particles showed the particles to be small and spherical within the size range of 450 nm to 7.25 μm. The majority of these particles lie within the size range that is optimal for pulmonary drug delivery *i.e.* ≤5 μm. Contrary to this, the large particles seen in Advair® Diskus® are the carrier lactose particles while the smaller particles are that of the drugs. The carrier particle is not intended to reach the lower respiratory tract; hence the size is always maintained to be ≥ 10 μm. The micronization process that is used to reduce the particle size of the drug to the respirable particle size range has great influence on the physical properties of the drug. In this case, the micronization technique used in Advair® manufacturing has rendered the drug particles into respirable non-spherical micro fines, while spray drying engineered the molecular mixture into spherical particles.

Another contributing factor to the morphology is the amorphous non-crystalline nature of the co-SD FP/SX/Man particles at the higher spray drying pump rates (*i.e.* 100% PR and 50% PR) and predominately amorphous (residual crystallinity) at the lowest pump rate (*i.e.* 25% PR) used in this study. Spray drying can make spherical particles which can be either crystalline or non-crystalline in the solid-state. Non-crystalline powders lack long-range molecular order becoming amorphous under certain spray drying conditions. Using advanced organic solution spray drying in closed-mode, it is evident from the lack of peaks in the XRPD diffractograms of co-SD FP/SX/Man particles at 50% PR and 100% PR that these are amorphous powders produced at medium and fast pump rates, respectively. The 25% PR particles produced at the slow pump rate show slight peaks suggesting residual crystallinity with predominantly amorphous character. This can be explained by the longer time allowed for alignment of crystals to occur during the slow spray drying process at 25% pump rate under these conditions. We have shown that advanced spray dried mannitol from organic solution retained its crystallinity at all pump rates (slow, medium, and high) through polymorphic interconversion using similar advanced organic solution spray drying in closed-mode conditions as a function of spray drying pump rate.^32,33,35^

On the other hand, SX which is known to exist in two polymorphic forms namely form I and form II has possibly undergone a polymorphic conversion from the stable form I to form II. The Raman spectra of the raw SX possess the characteristic peaks of form I at wavenumbers 748, 830, 927, 1349, 1405 and 2958 cm^−1^.^29^ However, the co-SD powders did not show the characteristic absorption bands of SX, which could be due to the fact that SD might have caused SX form I to convert to form II. Alternately, the quantity of SX in the co-SD powder is much less than FP, which could have subdued the SX peaks. Regarding the polymorphic transition, the X-ray diffraction pattern seen in Fig. 4 is consistent with the observation of ATR-FTIR peaks. There are two known polymorphs of FP; however, spray drying appears to have caused disruption to the crystal structure rendering it amorphous. The decreased residual water content in the co-SD FP/SX/Man particles is a good indicator of the physical stability of the particles. This suggests a diminished tendency to absorb moisture from the atmosphere due to capillary forces, which can lead to poor aerosol dispersion and polymorphic conversion. It was reported that SX–lactose binary mixtures have shown a decrease in aerosol dispersion due to enhanced capillarity.^50^

Chemical analytical techniques performed on the co-SD powders confirm the presence of both the drugs FP and SX. The prominent peaks of fluorine and sulfur atoms in the EDX spectra, the absorption bands of the IR and Raman spectra for “molecular fingerprinting” are molecular evidence for the presence of FP in all three co-SD powders as they are. However, the SX peaks were difficult to quantify. It has been previously reported that fluorescence of SX makes it harder to see the Raman shifts of polymorphic form II while using 514 nm laser excitation.^46^ Another possibility for lack of SX peaks is that the ratio of SX to FP was 1 : 5 making possibly below the limit of detection by these analytical methods. Raman mapping showed uniform distribution of FP in all the co-SD samples. Hence, HPLC was performed to identify and quantify the amount of each drug present in the co-SD FP/SX/Man particles at all the three spray drying pump rates. The combined chemical analyses indicate the presence of both drugs in the final co-SD formulations at all three spray drying pump rates.

The *in vitro* aerosol performance of the newly formulated co-SD FP/SX/Man powders were characterized with an inertial impactor to compare its aerosol performance with Advair® Diskus® particles. The Diskus® inhaler device has an aluminum strip prefilled with the powder formulation (Advair®). It is not possible to fill the Diskus® device with a powder formulation in a standard academic laboratory setting. Hence, a unit-dose capsule-based FDA-approved human inhaler device, the Handihaler®, was used.

The co-SD FP/SX/Man (100% PR) demonstrated higher FPF and lower MMAD values. The FPF of Advair® Diskus® formulation (*i.e.* an interactive physical mixture of respirable particles of jet-milled drugs physically blended with non-respirable large lactose carrier particles) cannot be calculated since it was not possible to measure the ED from this device. It is reported in the product package insert of Advair® Diskus® that 233 μg of FP and 45 μg of salmeterol base were delivered from the device when tested at 60 L min^−1^ for 2 seconds of actuation. However, it is unclear as to how much of this mass constitutes the fine particle mass (≤4.46 μm). Hence, the computation of FPF and ED of Advair® was not compared. The total mass of Advair® Diskus® particles deposited on NGI stages 2–7 was calculated to be 3.22 mg (after 3 actuations), which includes the fine particles of the lactose carrier. From Table 3, it is clear that the FPD of the co-SD powders is about ten times higher than that of the Advair® particles. Hence, co-spray drying with mannitol and using Handihaler® device can generate a higher fine particle mass of FP/SX. The statistical analysis showed that there was statistically significant difference between the FPD of all co-SD FP/SX/Man particles and Advair® (*P* value < 0.001). Similarly, the NGI stage deposition of the particles also showed significant difference between the co-SD FP/SX/Man particles and Advair® (*P* value < 0.01) physically blended particles. Yet, on comparing the aerosol performance of the three different co-SD particles, there was no significant difference between the 25% and 50% particles.

Previously, a comparative aerosol performance study of SX blended as binary and ternary mixtures with sugars such as lactose, glucose, mannitol and sorbitol was reported.^51^ In that study it was observed that mannitol and glucose had the highest FPF values as binary mixture with reduced moisture content and decreased particle adhesion. The results are similar to what is observed in this study; however, it is important to note that co-SD FP/SX/Man powders created molecular dispersions as opposed to an interactive physical blended mixture. Another study reported co-SD FP/SX with several excipients that had improved FPF using HPMC and polysorbate 80.^44^ HPMC and polysorbate 80 are common pharmaceutical excipients.

In other pulmonary diseases, mannitol (Bronchitol®) is newly indicated treatment in cystic fibrosis *via* inhalation. This study also showed that a molecular mixture with mannitol exhibited a superior aerosol performance. Considering the mucolytic action of mannitol and its ability to improve aerosol performance, it only seems appropriate to use it as excipient for diseases like asthma and COPD where excessive mucus production is noted.

## Conclusions

This study has successfully designed and generated lactose-free dry powder formulations containing the two drugs, FP/SX, molecularly mixed with mannitol as an excipient using advanced organic solution spray drying particle engineering design. Furthermore, the resulting co-SD powders had the properties essential for respiratory drug delivery. Solid-state characterization of the formulations demonstrated that the dry powder formulations are suitable for pulmonary drug delivery with enhanced aerosol performance as molecular mixtures. In addition, these respirable formulations comprised of solid-state nanoparticles/microparticles with aerosol properties conducive to targeting the mid- and deep lung regions. All powders aerosolized readily with high dispersion performance as mostly amorphous powders. Among the three different spray drying conditions reported in this study, 100% pump rate was identified to produce improved particles that had lower MMAD and higher FPF. Hence, dry powder inhaler formulations such as these can be used to treat asthma and COPD in patients with lactose allergy.

## Conflicts of interest

There are no conflicts to declare.

## Supplementary Material
